# "Someone told me": Preemptive reputation protection in communication

**DOI:** 10.1371/journal.pone.0200883

**Published:** 2019-04-24

**Authors:** Francesca Giardini, Stanka A. Fitneva, Anne Tamm

**Affiliations:** 1 Department of Sociology, University of Groningen, Groningen, The Netherlands; 2 Department of Psychology, Queen’s University, Kingston, ON, Canada; 3 Károli Gáspár University of the Reformed Church in Hungary, Budapest, Hungary; Arizona State University, UNITED STATES

## Abstract

Information sharing can be regarded as a form of cooperative behavior protected by the work of a reputation system. Yet, deception in communication is common. The research examined the possibility that speakers use epistemic markers to preempt being seen as uncooperative even though they in fact are. Epistemic markers convey the speakers’ certainty and involvement in the acquisition of the information. When speakers present a lie as indirectly acquired or uncertain, they gain if the lie is believed and likely do not suffer if it is discovered. In our study, speakers of English and Italian (where epistemic markers were presented lexically) and of Estonian and Turkish (where they were presented grammatically through evidentials) had to imagine being a speaker in a conversation and choose a response to a question. The response options varied 1) the truth of the part of the response addressing the question at issue and 2) whether the epistemic marker indicated that the speaker had acquired the information directly or indirectly. Across languages, if participants chose to tell a lie, they were likely to present it with an indirect epistemic marker, thus providing evidence for preemptive action accompanying uncooperative behavior. For English and Italian participants, this preemptive action depended respectively on resource availability and relationship with the addressee, suggesting cultural variability in the circumstances that trigger it.

*“And if*, *to be sure*, *sometimes you need to conceal a fact with words*, *do it in such a way that it does not become known*, *or*, *if it does become known*, *that you have a ready and quick defense*.*”*

*-*Niccolò Machiavelli, 1522

## Introduction

The opportunities for information sharing afforded by the emergence of language were of pivotal importance in human evolution [1,2]. Information sharing was essential for cooperative problem solving, such as food foraging, where interdependent group members needed to coordinate their efforts. Information sharing was also essential for the increase of group size, because it enabled exchange of information about others, thus allowing a powerful reputation system to develop that does not depend on direct observation. This reputation system likely explains the ubiquity of information sharing we observe today: from situations where the costs to speakers are minimal, e.g., providing tourists with driving directions and reviewing online restaurants and books, to ones where information sharing appears truly altruistic as it gives access to limited and valuable resources and entails high costs for speakers, e.g., sharing information about a competitive grant. In all of these cases, the goal to obtain or maintain positive reputation motivates speakers to share information and deters them from withholding information and lying.

Theories of cooperation explain information sharing by highlighting the role of reputation in systems based on indirect reciprocity. Individuals cooperate with the expectation that their good behavior will be rewarded with positive reputation and cooperation not just by the recipient of their action but by others as well [3]. In a “market for cooperators” [4], or when partner choice is available, individuals may compete for the most altruistic partners and non-altruists may become ostracized [5–7]. In other words, speakers who do not share information or lie may be avoided and have difficulty finding partners when in need. Thus, reputational concerns can explain why speakers assume not only the relatively small costs of giving directions to a tourist but also the larger costs inherent in situations when their interests compete with those of addressees. Theories of impression management, developed in psychology and sociology, similarly emphasize that human actions are partly driven by the intention to elicit positive evaluation from partners [8–11]. In support of these positions, extensive evidence now shows that both adults and children engage in more cooperative behavior when selfish actions are observable and reputational concerns are higher, suggesting that individuals actively engage in reputation protection [12–16]. The pressures of group living may have indeed led to the evolution of psychological regulatory mechanisms for tracking one’s social valuation [17].

Yet, deception is part of daily life [18–20]. Deception is a psychological process by which one individual deliberately attempts to convince another person to accept as true what he or she knows to be false, with the aim to gain some type of benefit or to avoid loss [21]. Lies are frequent in daily interpersonal interactions [22], in online reviews [23], and even in controlled laboratory experiments [24]. By some estimates, as much as 26% of interpersonal communicative interactions involve deception [20]. Explanations range from population equilibria of cooperation being robust to some incidence of uncooperative behavior [25] to low reputational costs of deception because, for example, it is difficult to detect [26,27]. Still, the incidence of deception suggests that speakers fairly often prioritize their own interests over the interests of others, and thus the possibility that reputation is not as strong a constraint on information sharing as theorized. In this paper we suggest that this is not the case. Through language, individuals have greater control than implied by the choice between veracity and deception, the former with potentially high economic costs and the latter with potentially high reputational costs. Specifically, we suggest that individuals deploy preemptive reputation protection strategies that allow them to be deceptive but avoid the reputation-damaging consequences of deception.

One way in which preemptive reputation protection is possible is through the use of epistemic markers. Their role is based on the information they convey on the one hand, and on the discourse status of this information on the other hand. To ground the discussion, consider the following exchange in the context of both A and S needing childcare services and S having found that the Blue Center is better than the Red Center. In the example, S lies. The epistemic markers are italicized.

A: Which childcare center is better?

S: *I don’t know exactly*, *but someone told* me that the Red Center is better. You should try to register your kids there.

Informationally, epistemic markers convey the speaker’s qualification of some other information the speaker presents. For instance, speakers may qualify information by conveying their certainty, e.g., “I don’t know exactly …”. Or they may or may not present themselves as the source of the information (“someone told me …” or “I saw …”). In some languages, such as Turkish and Estonian, the source of information can be presented not just through lexical expressions but also through grammatical elements called evidentials.

At the level of discourse, a speaker’s response to a question can be divided into presenting “at-issue” and “not-at-issue” content [28]. Information is “at-issue” if it directly addresses the question or problem at hand. In the exchange above, A’s question defines the issue as daycare quality. Thus, the at-issue part of the S’s response is “the Red Center is better”. The epistemic expression “I don’t know exactly, but someone told me” is part of the response that is not at issue. Epistemic markers can provide at-issue information, e.g., when emphasized, as in “I *saw* the Red center”, and when offered in response to questions such as “How do you know?” Most often, however, as in the exchange above, this information is provided as a backgrounded parenthetical comment [29,30].

Epistemic markers are relevant to reputation protection because through them speakers can manipulate the distance between themselves and the information. Similarly to the makers of any product offered to others, speakers bear greater responsibility for what they say if they are its source or if they vouch for it. If they present information as originating with someone else, they are distancing themselves from it, thus reducing their responsibility. Furthermore, because the epistemic information is not-at-issue, it is less likely to be scrutinized (e.g., by asking: “Who exactly told you?”). Thus, manipulating the epistemic markers poses fewer risks to the speakers’ reputations than manipulating at-issue information.

In sum, if speakers choose to lie, they can also preemptively distance themselves from the lie in case it is discovered through the use of epistemic markers. The use of epistemic markers thus may represent in Machiavelli’s words thinking ahead about “a ready and quick defense.” They may protect speakers’ reputations as cooperative informants even if, later on, addressees discover that they had lied.

The goal of the present research was to examine if speakers indeed distance themselves from false information via manipulating its epistemic status. Research on deception shows that expressing uncertainty, also called hedging, is associated with deceptive information in both face-to-face and online settings [31–33]. However, to date there are few experimental investigations of whether speakers indeed use epistemic markers to balance the pressures of cooperation and self-interest, and whether they take into account elements like their relationship with the addressee or the presence of competition for resources. Furthermore, as the research has been based on English corpora, it is not clear whether the same results will be observed across languages.

We examined whether speakers of four languages–English, Estonian, Italian, and Turkish–were more likely to present information that they had acquired directly as uncertain and/or indirect when falsifying it than when truthfully reporting it. We designed the studies so that in some conditions, participants are inclined to convey the truth, and in other conditions they are inclined to lie. Specifically, they were presented with situations where a Speaker gained first-hand information about a resource and then another actor, the Addressee, asked about it. (We use Speaker and Addressee to refer to the roles played in the situation.) The resource was either limited or amply available and the Addressee was either a friend or an acquaintance of the Speaker. Prior research shows that competition over resources and lower expectations for reencounter decrease cooperation and increase the likelihood of lying [34]. Participants were asked to put themselves in the shoes of the Speaker, i.e., someone who gained first-hand experience about a resource, and choose one of four possible answers to a question about that resource. They were asked to imagine a communicative situation and behavior, and thus we refer to them as “speakers,” but they did not actually produce spoken language.

All four alternatives participants chose among conveyed relevant information and were appropriate in the situation. Participants were asked to choose the answer they were more likely to give (see S1 Appendix), so there were no right or wrong choices. The at-issue part of the answers (directly addressing the question) was either true (the Blue Center is better) or false (the Red Center is better). In addition, the answers varied the not-at-issue content introduced through an epistemic marker: whether the information was presented as originating in the self or as originating in someone else and being indirect. Of primary interest was whether the latter, indirect, epistemic markers would be selected more often when participants chose to respond falsely to the question.

As the four studies employed similar methods, we combine the presentation of their methods and results.

## Methods

### Participants

Participants were recruited from major universities and surrounding urban communities in Canada, Italy, Estonia, and Turkey through posts on listserves and social media sites. Potential participants were given a link to SurveyMonkey (www.surveymonkey.com) where they could complete the study. To participate in the study, individuals had to identify themselves as native and monolingual speakers of the target languages (for Canada, English). Information about the final samples is provided in Table 1. The Canadian and Turkish participants received course credit or had the opportunity to enter into a $25 draw as compensation.

**Table 1:** Characteristics of the samples in the four studies.

	English	Italian	Turkish	Estonian
Years of data collection	2013–2014	2014	2013	2013, 2015–2016
N	80	104	346	220
Mean age (range)	24.9 (18–59)	34.4 (19–67)	22.3 ^a^	37.3^b^ (18–77)
% female (number reporting gender)	73% (79)	68% (101)	65% (345)	74% (213)

Note

^a^ based on the 63% of participants who reported exact age

^b^ participants either responded with year of birth or a range; in the latter case, the midpoint of the range was used to calculate the average

### Sample size

Sample size for the Canadian and Italian studies was arbitrarily set to a minimum of 80 and a stop rule was the end of the term in which the studies were being conducted. The target sample size range for the Turkish and Estonian studies, which followed a different design, was tentatively set to 230–350 based on recommendations for logistic regression [35]. Additionally, the sample sizes for these studies depended on the number of participants needed for the larger batteries the studies were part of.

A final sample of 80 was achieved in the Canadian study. One participant was replaced due to taking over a day to answer and three more for not answering all items. The final sample of the Italian study consisted of 104 respondents, and there were no exclusions. The final sample size for the Turkish study included 346 participants. Participants were not included in the analyses if they accessed the survey twice (7) or failed to answer (21). The final sample size for the Estonian study included 220 participants. Here, the data were collected in two rounds, as the first round resulted in only 124 participants. Eleven participants were not included as they failed to answer.

### Materials

English and Italian speakers were presented with four scenarios (see S1 Appendix for the English version), and Estonian and Turkish speakers with just one (the Daycare scenario) because the study was included in a longer testing session, during which participants completed a set of unrelated other tasks. All scenarios had similar structure. An actor, the Speaker, gained first-hand information about a resource: quality of daycare centers, a foundation offering scholarships, a cheap used car dealership, or a weekend concert. Then a second actor, the Addressee, with interests in the resource, requested information about it. Each scenario had four versions based on the factorial manipulation of resource availability (ample vs. limited) and the relationship between the actors (friends vs. acquaintances). For example, in the Daycare scenario introduced above, the Speaker is looking for a daycare for her child and finds out that the Blue Daycare Center is better than the Red Daycare Center. The (better) Blue Center either had lots of openings or could accept only a few children. The Addressee, who has twins, is also looking for daycare. To vary relationship, the Addressee was introduced either as a “close friend” or just by name or as someone the Speaker had met once.

After the introduction of the Speaker, the Addressee, and the Addressee’s information request, participants were asked to imagine being the Speaker and select an answer. The four answers participants were asked to choose among were created by crossing the at-issue content i.e., the part of the answer that addressed the information request, and two epistemic markers. The at-issue content was either true or false. For example, in the Daycare scenario, two of the answers stated that the Blue Center was better (true), and two that the Red Center is better (false).

Table 2 shows the epistemic markers used in the four studies. The studies sampled epistemic information of different kinds and different grammatical status. Across studies, the epistemic markers conveyed either direct evidence and certainty (*self* column in Table 2) or indirect evidence and uncertainty (*indirect* column in Table 2). In English and Italian, the epistemic markers were lexical expressions, specifically descriptions of the experience that has led to information acquisition (e.g., visiting daycares) or “I don’t know exactly but someone told me.” In Estonian and Turkish, the studies drew on the grammaticalized evidential systems of these languages. Estonian has an optional indirect evidential–*vat* that indicates that the information is hearsay and, as in many other languages, directly attested information is unmarked [36]. In Turkish, there are two evidential markers: -*Di* for first-hand evidence and -*mI*ş for indirect (second-hand or inferential) evidence. They are mandatory for past tense statements. The present research used statements in the present tense, however, in which the direct evidential is not realized [37]. Thus, as in Estonian, the directly attested information in Turkish was unmarked. The general pragmatic inference for evidentially unmarked sentences is that the information is directly acquired by the speaker [38].

**Table 2:** Epistemic markers used in the response options in the four studies.

	Epistemic Markers	
	Self	Indirect
Lexical epistemic markers		
English	*I visited both daycares* [and the Blue Center is definitely better.] You should try to register your kids there.	*I do not know exactly*, *but someone told me* that [the Blue Center is definitely better.] You should try to register your kids there.
Italian	*Ho visitato tutti e due i centri* [ed il Centro Blu è decisamente migliore.] Dovresti provare ad iscrivere i gemelli lì.	*Non saprei esattamente*, *ma qualcuno mi ha detto* che [il Centro Rosso è migliore.] Dovresti provare ad iscrivere i gemelli lì
Grammatical epistemic markers		
Estonian	[Sinine Lasteaed] on [parem].^1^	[Sinine Lasteaed] ole*vat* [parem].
Turkish	[Mavi Yuva daha] iyi.^1^	[Mavi Yuva daha] iyiy*miş*.

*Note*. Epistemic markers are italicized. The at-issue content of the responses, here addressing the request for daycare center information, is shown in square bracket […]. The at-issue content could be either true or false.

^1^In Estonian and Turkish unmarked present indicative sentences were used to convey direct acquisition of the information as Estonian does not have a direct evidential, and in Turkish the direct evidential is only realized in the past tense.

### Design and procedure

For the English and Italian studies, the four versions of each scenario were distributed across four lists. Each list contained each scenario and represented the four conditions resulting from the crossing of the factors of resource availability and relationship between the speaker and the addressee. Approximately an equal number of English and Italian speakers were presented with each list. For the Estonian and Turkish studies, participants were presented with one of the four versions of the Daycare scenario. About an equal number of participants responded to each version.

All participants completed the study online on SurveyMonkey (www.surveymonkey.com) after providing informed consent. The consent form informed the participants that they have to answer a question after reading a short vignette /vignettes. For English and Italian participants, the four scenarios were presented in a fixed order (see S1 Appendix). The four answers available to participants to choose among were also presented in a fixed order: true at-issue content/self epistemic marker, false at-issue content/self epistemic marker, false at-issue content/indirect epistemic marker, true at-issue content/indirect epistemic marker. The text of each scenario stayed on the screen until participants provided a response to avoid memory issues.

## Results

We begin by reporting the distribution of the answers selected by participants in each study. After that, we focus on the correlates of participants’ choice of epistemic markers.

### Answer distribution

**Fig 1:**
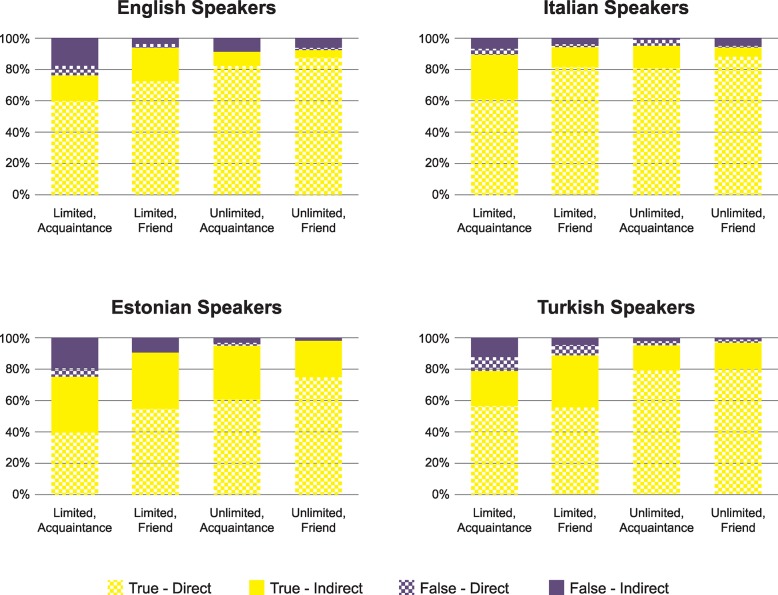
Distribution of the four answers in each condition in each of the four studies. Condition was varied within subject for Canadian and Italian speakers and between subject in Estonian and Turkish speakers.

Fig 1 shows the distribution of responses in each condition of the four studies. Condition here is defined by whether communication was about limited or unlimited resources and whether information was transmitted to an acquaintance or a friend. As Fig 1 shows, in all conditions and across languages, participants selected answers that present truthful at-issue content and indicate that the Speaker has direct evidence above chance. On average, the at-issue content was truthfully reported by English speakers 89% of the time (*t*(79) = 19.717, *p* < . 001; median = 1, Wilcoxon signed rank test *Z =* 7.783, *p* < . 001), by Italian speakers 93% of the time (*t*(103) = 30.515, *p* < . 001; median = 1, Wilcoxon signed rank test *Z =* 9.302, *p* < . 001), by Estonian speakers 90% of the time (*χ*^2^ (1, N = 220) = 136.04, *p* < . 001), and by Turkish speakers 90% of the time (*χ*^2^ (1, N = 346) = 221.76, *p* < . 001). On average, the self epistemic marker was chosen by English speakers 79% of the time (*t*(79) = 11.07, *p* < .001; median = .75, Wilcoxon signed rank test *Z =* 6.736, *p* < . 001), by Italian speakers 80% of the time (*t*(103) = 13.965, *p* < . 001; median = .75, Wilcoxon signed rank test *Z =* 7.915, *p* < . 001), by Estonian speakers 59% of the time (*χ*^2^ (1, N = 124) = 6.92, *p* = .009), and by Turkish speakers 73% of the time (*χ*^2^ (1, N = 346) = 73.06, *p* < . 001).

The analyses of the determinants of the truthfulness of the at-issue content are available in the supplementary materials (see S1 Table). The sensitivity of deception rates to the manipulation of Resource Availability and Relationship between the Speaker and the Addressee varied across studies (see supplemental materials, S1 Table). However, for the analyses of epistemic marker choice, it is important that deception rates were similar across languages.

## Analytical approach

Of key interest in the present research was whether speakers varied epistemic markers as a function of the truthfulness of the at-issue content. As we had repeated binary measures data in English and Italian, the choice of an epistemic marker was modeled using generalized estimating equations (GEE) which can be seen as an extension of logistic regression [39,40]. The use of an indirect epistemic marker was modeled using a binomial distribution with a logit link-function. The repeated data in English and Italian were modeled using several correlation structures. Based on information criteria and computational convergence, an independent correlation matrix was selected for the models reported here.

In addition to the truthfulness of the at-issue content, the GEE models included resource availability, relationship with the addressee, and the interactions among the three factors. Even though epistemic markers generally contribute not-at-issue content, as pointed out in the Introduction, in some circumstances they can have at-issue status. Their manipulation thus can be seen as lying. If so, speakers’ decisions about marker use may be affected by resource availability and their relationship with the addressee [18]. Although we did not have specific predictions, we reasoned that an effect of these variables is more likely when epistemic markers are lexical than grammatical (i.e., in the English and Italian studies) as lexicalization is correlated to having an at-issue status [29].

Details of the model selection process are presented in S2 Table. The three-way interaction between truthfulness, resource availability, and relationship did not lead to improvement of the models and did not have theoretical interest, so it was not included. Initial analyses showed that scenario did not enter in any interactions in the data from English and Italian speakers, thus it was included as a simple effect. Preliminary analyses also explored the effect of gender. As none was found, this variable was not considered further either.

### Epistemic marker choice

Table 3 summarizes the results from the four studies. All four studies showed that participants, when asked to adopt the role of speakers who have valuable information, chose an epistemic marker in a tight relation with whether they decided to answer the Addressee’s question truthfully or lie. In all studies, the at-issue content was more likely to be presented as indirect when it was false. For English, Italian, Turkish, and Estonian, the odds of presenting information as indirect were 21.4 (95% CI: 8.1–56.3), 7.8 (95% CI: 3.5–17.3), 3.1 (95% CI: 1.5–6.4), and 8.43 (95% CI: 2.76–25.75) times larger respectively if the information was falsified than if it was true. (Odds ratios are reported from the models including truthfulness only because parameter estimates from the complete models including interactions are relative to the other terms.)

**Table 3:** Selection of epistemic marker as a function of truthfulness of the at-issue content, resource availability, and relationship with the addressee.

	Language of Participants			
Effect	English (N = 80)	Italian (N = 104)	Turkish (N = 346)	Estonian (N = 220)
Truthfulness	38.736***	18.013***	4.198*	7.591**
Resource Availability	.099	4.872*	1.765	.847
Relationship	1.196	.531	.587	1.328
Truthfulness X Resource Availability	6.176*	0	.279	.002
Truthfulness x Relationship	.539	9.757**	1.899	N/A^b^
Resource Availability X Relationship	1.500	.968	.386	.334
Scenario	11.411**	20.391***	N/A ^a^	N/A ^a^

*Note*: Wald chi-square tests significance levels associated with the factors in the GEE models:

*** < .001

** < .01

* < .05

a Only one scenario was used in Estonia and Turkey, so the term was omitted.

^b^ Term was omitted because of separation in the data: Estonian speakers always used an indirect evidential when lying to a friend.

While the effect of truthfulness of the at-issue content was robust in all four studies, the effects of resource availability and relationship between the Speaker and Addressee varied. For English participants, resource availability modulated the effect of truthfulness. Even though the effect of truthfulness was evident both when resources were ample and limited, the effect, as indicated by the odds ratio, was stronger in situations with ample resources (OR 8.6 vs. 148). In other words, if English participants lied when the resources were ample, they were even more likely to present the information with an indirect epistemic marker than if they lied when the resources were limited.

Italian participants, on the other hand, were more likely to present the at-issue information as originating in them (i.e., choose a self epistemic marker) when resources were ample, regardless of the truthfulness of the response. Of greater interest, in this study relationship modulated the effect of truthfulness on choice of epistemic marker. Even though the effect of truthfulness was evident both when Italian participants shared information with a friend and an acquaintance, the effect was stronger when talking with a friend. The odds of selecting an indirect epistemic marker when lying to a friend were 27.9 compared to 3.3 when lying to an acquaintance.

As indicated in Table 3, in the Estonian study, the interaction effect of relationship and truthfulness of the at-issue content on the choice of epistemic marker could not be numerically estimated. Estonian participants *always* chose an indirect epistemic marker when lying to a friend. This suggests that relationship also has considerable influence on Estonian participants’ choice of epistemic markers when they lie. The Turkish study was the only one where neither resource availability nor relationship with the addressee constrained the participants’ choice of epistemic marker.

In the English and Italian studies, we also observed an effect of Scenario. In both studies, the Scholarship scenario elicited the greatest use of indirect epistemic marker (about 35%), followed by the Daycare scenario (used also in the Estonian and Turkish studies, about 20%), and then the Car and Concert scenarios, which were about the same (15–11%). While the effect of Scenario suggests that the situation can affect the likelihood of an indirect epistemic marker use, as noted in the Analytical Approach section, Scenario did not modulate the effects of any of the other variables. This gives us confidence that, even though only the Daycare scenario was used in the Estonian and Turkish studies, the results from these studies generalize to other situations.

## Discussion

The present research provides experimental evidence for a novel kind of link between the way information is reported and the human reputation management system. In particular, forecasting of the effects of behavior on reputation appears to involve not only context-sensitive choices such as being more cooperative when re-encounters are likely [18,34,41], but also the preemption of reputational repercussions when choosing not to cooperate. In four studies involving four different languages, we found that when participants opted for a lie–a paradigmatic uncooperative behavior–they presented it with an epistemic marker, thus actively engaging in preemptive reputation protection.

In the tradition of self-presentation and impression management research human actions such as information sharing and deception reflect a foresight into the reputational consequences of behavior [8–11]. Reputation is a powerful motivation for a whole range of actions (for a multi-disciplinary overview, see [42]), and an effective reputation management system requires different competences. Here, we suggest that language, more specifically the selection of epistemic markers might be part of the repertoire of reputation management. The present research demonstrates that, as speakers, humans provide information that may frame their actions for the addressees, thus constraining addressees’ inferences and the possible reputational consequences of the actions. In other words, speakers do not only modify their own behavior as a result of reputational pressures but attempt to manipulate the reputational judgment that their (sometimes self-serving) behavior may elicit. Presenting information as uncertain and/or indirectly acquired distances individuals from the information, which reduces their responsibility and mitigates the reputational consequences of deception.

Reputation management involves a cognitive mechanism that allows individuals to forecast how their reputations will be affected by their actions and act accordingly [41]. This mechanism is engaged when people consider the gains and risks of lying [10,43]. We propose that it also incorporates the computation of preemptive action when the path of uncooperative behavior is chosen. Such a system is consistent with proposals that human psychology has evolved regulatory mechanisms that track and respond to one’s social valuation [17]. For example, emotions such as shame, guilt, and anger can be seen as neurocomputational responses to threats of perceived social devaluation [44]. The cognitive reputation management mechanism is expressed in many ways in linguistic behavior. Speech acts like apologies are reactions to discovered transgressions aiming to restore one’s reputation [45,46]. Seemingly puzzling acts like spontaneous confessions are mostly observed when they will not threaten one’s reputation or when the likelihood of discovering the speaker’s transgression is high [47]. Language also provides means of changing the payoffs in information exchanges, e.g., through indirectness (40). Finally, as we show, humans use linguistic devices to preemptively protect their reputation when behaving uncooperatively.

While the planning and execution of a preemptive linguistic action may be rooted in an evolved reputation management system, this action relies on acquired linguistic and pragmatic knowledge, including the acquisition of evidentials and social norms. Thus, deploying a preemptive action may be effortful and constrained by cognitive resources. The advantage of being preemptive is greater control over one’s reputation. If an addressee discovers that the speaker has been deceptive, the spread of this potentially reputation damaging information is difficult to control [48]: speakers may not be at the right place at the right time to defend themselves, and they may not reach everyone who has been reached by the information. A preemptive action, such as adding an indirect epistemic marker to the at-issue information, may shield a speaker as it provides a context in which the addressee can interpret the false information, and the speaker may avoid being blamed altogether. Thus, being preemptive may be well worth any cognitive effort it implies.

Information sharing often appears altruistic as it involves effort and no obvious benefits, e.g., when we help out a tourist or review a product online. Perhaps as a consequence, it has been predominantly examined experimentally in conditions where the speaker’s self-interest leads to no immediate benefits and minimal costs [49,50]. This, however, should not obscure the fact that information sharing has costs. Many societies have evolved to collectively assume some of these costs, as in the case of publicly funded education. When information sharing is costly for individuals, they can be expected to deceive or conceal information while taking steps to protect their reputation. The present study provides a demonstration of such Machiavellianism. It can be also observed in gossip—the sharing of evaluative information about an absent third party [51]. When gossiping, hedging the statements with epistemic qualifications such as “someone told me” or “rumor has it” helps spread false or uncertain information without the risk of being punished by both actors, the addressee and the gossip target [52]. Not surprisingly, indirect evidentials are prominent in gossip situations [53,54].

Both certainty and source markers are sometimes discussed as bearing on the reliability of information [55]. The speaker’s presentation of at-issue information as indirectly acquired might make the information appear less reliable, thereby discouraging the addressee from using it. However, the experimental evidence supporting the reliability interpretation of epistemic tools comes from examining receivers’ attitudes [56–58]. The present data cannot be explained by assuming that participants manipulated epistemic status in order to manipulate the reliability of information. If this were the case, in conditions of competition, participants should be more likely to present true than false information as indirect, thus discouraging the use of the former but not of the latter. None of the four studies, however, showed this pattern (see Fig 1).

A major limitation of this present research is that participants were asked to answer questions about hypothetical situations. They were not involved in actual conversation, and therefore it is not possible to assess the extent to which selecting among predefined answers corresponds to active, online decisions about lying or telling the truth. Furthermore, in conversation, individuals can answer in a large number of ways including laughing, changing topic, or saying “I don’t know”. The participants in our study did not have this range of options. However, the answers they chose among were all relevant [59,60]. The use of hypothetical situations and predefined answers were necessary for the present research, for which it was crucial to distinguish between true and false content. Further research is needed to examine whether the findings generalize to more naturalistic settings.

Although across all languages participants distanced themselves from false information by presenting it as indirect, the results of the studies differed both in terms of the magnitude of the effect of truthfulness and in terms of how truthfulness interacted with the other variables. For example, we found that English participants were more likely to use an indirect epistemic marker when presenting false information about an ample resource than about a limited resource. And Italian and Estonian participants were more likely to use an indirect epistemic marker when presenting false information to a friend than to an acquaintance. Of course, lying to a friend has worse consequences for one’s reputation than lying to an acquaintance, which may lead to greater tendency to preemptively protect oneself. Similarly, lying when resources are ample is hard to explain, which may raise the stakes for reputation and lead speakers to introducing distance between themselves and their lie. However, this reasoning clearly did not generalize across all languages.

Identifying the reasons for the differences among the studies goes beyond the scope of the present research. To some extent they may be due to the methodological differences among the studies and the different realization of epistemic markers. For example, the Estonian and Turkish samples were exposed only to the Daycare scenario. As a reminder, Scenario did not interact with the other variables in the analyses of the English and Italian data, but the generalizability of our findings will be boosted by examining a wider range of situations in *all* languages. In addition, as noted, lexicalization may be correlated to information having at-issue status [49], and the influence of resource availability and relationship between the speaker and the addressee may be constrained to at-issue content. In line with this suggestion, Turkish was the only language with a mandatory grammaticalized evidential system in this set of studies, and here resource availability and relationship had no effect. However, given that the results of the English and Italian studies differed despite a close methodological match, the causes of the differences in results, if replicated, would suggest factors other than methods and semantics of the epistemic markers. Further research should be undertaken to understand the conditions favoring preemptive reputation protection and to what extent they depend on cultural and linguistic differences.

A potentially intriguing aspect of our results is the lack of gender differences. There is a growing literature showing that men and women behave differently depending on the kind of lying. Capraro [61] shows that males are more likely to use black lies, e.g., lies that benefit the liar at a cost for another person, and altruistic white lies. In our research, as resource availability and the relationship between the Speaker and the Addressee varied across conditions, mis-information was not consistently a white or a black lie and our studies may have been underpowered to detect an effect of gender. In addition, in some conditions, it is not straightforward to classify misinformation as a white or a black lie, e.g., when resources are ample. While there might be gender differences in lying, the evidence for gender differences in sensitivity to reputational factors is still scarce and requires further investigation [62].

It is important to extend the findings to other languages with grammaticalized evidential systems as well as to examine responses to different ways of lexicalizing epistemic markers in languages without such systems. Future research is also needed to examine the development of preemptive reputation protection. Children’s behavior shows sensitivity to reputational pressures by age five [13,41]. However, using language as a tool to avoid the consequences of lying may require further linguistic and cognitive maturation.

## Supporting information

S1 Appendix Four scenarios were used in English and Italian. Only the Daycare scenario was used in Estonian and Turkish. The scenarios were distributed into four lists according to a Latin Square design for English and Italian participant.
(DOCX)

S1 Table Effect of resource availability and relationship on content presentation based on GEE analyses. (DOCX)

S2 Table Model selection. (DOCX)

S1 Fig Proportion of answers with true content by resource availability and relationship with addressee. (DOCX)

## References

[1] Dunbar RI. Gossip in evolutionary perspective. Rev Gen Psychol. 2004;8(2):100.

[2] Tomasello M, Melis AP, Tennie C, Wyman E, Herrmann E. Two key steps in the evolution of human cooperation: the interdependence hypothesis. Curr Anthropol. 2012;53(6):673–92.

[3] Nowak MA, Sigmund K. Evolution of indirect reciprocity. Nature. 2005 10 27;437(7063):1291–8. 10.1038/nature04131 16251955

[4] Hammerstein P, Noë R. Biological trade and markets. Philos Trans R Soc Lond B Biol Sci. 2016 2 5;371(1687):20150101 10.1098/rstb.2015.0101 26729940 PMC4760201

[5] Milinski M. Reputation, a universal currency for human social interactions. Phil Trans R Soc B. 2016 2 5;371(1687):20150100 10.1098/rstb.2015.0100 26729939 PMC4760200

[6] Wu J, Balliet D, Van Lange PAM. Reputation management: Why and how gossip enhances generosity. Evol Hum Behav. 2016 5 1;37(3):193–201.

[7] Roberts G. Competitive altruism: from reciprocity to the handicap principle. Proc R Soc B Biol Sci. 1998 3 7;265(1394):427–31.

[8] Bromley DB. Reputation, image, and impression management New York: John Wiley & Sons; 1993.

[9] Goffman E. The presentation of self in everyday life. Garden City, NY: Doubleday & Co., Inc; 1959. (Doubleday Anchor Books).

[10] Leary MR, Kowalski RM. Impression management: A literature review and two-component model. Psychol Bull. 1990;107(1):34–47.

[11] Baumeister RF. A self-presentational view of social phenomena. Psychol Bull. 1982;91:3–26.

[12] Piazza J, Bering JM. Concerns about reputation via gossip promote generous allocations in an economic game. Evol Hum Behav. 2008 5 1;29(3):172–8.

[13] Ingram GPD, Bering JM. Children’s tattling: The reporting of everyday norm violations in preschool settings. Child Dev. 2010 5 1;81(3):945–57. 10.1111/j.1467-8624.2010.01444.x 20573115

[14] Bateson M, Nettle D, Roberts G. Cues of being watched enhance cooperation in a real-world setting. Biol Lett. 2006 9 22;2(3):412–4. 10.1098/rsbl.2006.0509 17148417 PMC1686213

[15] Sommerfeld RD, Krambeck H-J, Milinski M. Multiple gossip statements and their effect on reputation and trustworthiness. Proc R Soc B Biol Sci. 2008 11 7;275(1650):2529–36. 10.1098/rspb.2008.0762 PMC2603203 18664435

[16] Beersma B, Van Kleef GA. How the grapevine keeps you in line: gossip increases contributions to the group. Soc Psychol Personal Sci. 2011 11 1;2(6):642–9.

[17] Tooby J, Cosmides L, Sell A, Lieberman D, Sznycer D. Internal regulatory variables and the design of human motivation: A computational and evolutionary approach In: Elliot AJ, editor. Handbook of approach and avoidance motivation. Mahwah, NJ: Lawrence Erlbaum Associates; 2008 p. 251–71.

[18] DePaulo BM, Kashy DA. Everyday lies in close and casual relationships. J Pers Soc Psychol. 1998;74(1):63–79. 9457776 10.1037//0022-3514.74.1.63

[19] Hancock JT, Curry LE, Goorha S, Woodworth M. On lying and being lied to: A linguistic analysis of deception in computer-mediated communication. Discourse Process Multidiscip J. 2008;45(1):1–23.

[20] Hancock JT, Thom-Santelli J, Ritchie T. Deception and design: The impact of communication technology on lying behavior In: Proceedings of the SIGCHI Conference on Human Factors in Computing Systems [Internet]. New York, NY, USA: ACM; 2004 p. 129–134. (CHI ‘04).

[21] Abe N. How the Brain Shapes Deception: An Integrated Review of the Literature. The Neuroscientist. 2011 3 30;17(5):560–74. 10.1177/1073858410393359 21454323

[22] DePaulo BM, Kashy DA, Kirkendol SE, Wyer MM, Epstein JA. Lying in everyday life. J Pers Soc Psychol. 1996;70(5):979–95. 8656340

[23] Ott M, Cardie C, Hancock J. Estimating the Prevalence of Deception in Online Review Communities. In: Proceedings of the 21st International Conference on World Wide Web [Internet]. New York, NY, USA: ACM; 2012. p. 201–210. (WWW ‘12).

[24] Fischbacher U, Föllmi-Heusi F. Lies in Disguise—An Experimental Study on Cheating. J Eur Econ Assoc. 2013 6 1;11(3):525–47.

[25] Iñiguez G, Govezensky T, Dunbar R, Kaski K, Barrio RA. Effects of deception in social networks. Proc R Soc Lond B Biol Sci. 2014 9 7;281(1790):20141195. 10.1098/rspb.2014.1195 PMC4123708 25056625

[26] Ekman P, O’Sullivan M. Who can catch a liar? Am Psychol. 1991;46(9):913–20. 1958011 10.1037//0003-066x.46.9.913

[27] Vrij A, Granhag PA, Porter S. Pitfalls and opportunities in nonverbal and verbal lie detection. Psychol Sci Public Interest. 2010 12 1;11(3):89–121. 10.1177/1529100610390861 26168416

[28] Roberts C. Information structure: Towards an integrated formal theory of pragmatics. Semant Pragmat. 2012;5:1–69.

[29] Murray SE. The Semantics of Evidentials Oxford: Oxford University Press; 2017. 191 p.

[30] Simons M. Observations on embedding verbs, evidentiality, and presupposition. Lingua. 2007 6;117(6):1034–56.

[31] Buller DB, Burgoon JK. Deception: Strategic and nonstrategic communication In: Daly JA, Weimann JM, editors. Strategic interpersonal communication. Hillsdale, NJ: Erlbaum; 1994 p. 191–223.

[32] Ott M, Choi Y, Cardie C, Hancock JT. Finding deceptive opinion spam by any stretch of the imagination. In: Proceedings of the 49th Annual Meeting of the Association for Computational Linguistics: Human Language Technologies—Volume 1 [Internet]. Stroudsburg, PA, USA: Association for Computational Linguistics; 2011 [cited 2017 Oct 16]. p. 309–319.

[33] Newman ML, Pennebaker JW, Berry DS, Richards JM. Lying words: predicting deception from linguistic styles. Pers Soc Psychol Bull. 2003 5 1;29(5):665–75. 10.1177/0146167203029005010 15272998

[34] Krasnow MM, Delton AW, Tooby J, Cosmides L. Meeting now suggests we will meet again: Implications for debates on the evolution of cooperation. Sci Rep. 2013 4 29;3:srep01747. 10.1038/srep01747 PMC3638167 23624437

[35] Peduzzi P, Concato J, Kemper E, Holford TR, Feinstein AR. A simulation study of the number of events per variable in logistic regression analysis. J Clin Epidemiol. 1996 12;49(12):1373–9. 8970487 10.1016/s0895-4356(96)00236-3

[36] Tamm A. The Estonian partitive evidential: Some notes on the semantic parallels between the aspect and evidential categories In: Hogeweg L, de Hoop H, Mal’chukov A, editors. Papers from TAM TAM: Cross-linguistic semantics of Tense, Aspect, and Modality. Amsterdam: John Benjamins; 2009 p. 365–401.

[37] Johanson L. Evidentiality in Turkic In: Aikhenvald A, Dixon R, editors. Studies in evidentiality. Amsterdam & Philadelphia: John Benjamins; 2003 p. 273–90.

[38] Lazard G. On the grammaticalization of evidentiality. J Pragmat. 2001 3 1;33(3):359–67.

[39] Hardin JW, Hilbe JM. Generalized Estimating Equations: Introduction In: Wiley StatsRef: Statistics Reference Online [Internet]. American Cancer Society; 2014

[40] Liang K-Y, Zeger SL. Longitudinal data analysis using generalized linear models. Biometrika. 1986;73(1):13–22.

[41] Engelmann JM, Over H, Herrmann E, Tomasello M. Young children care more about their reputation with ingroup members and potential reciprocators. Dev Sci. 2013 11 1;16(6):952–8. 10.1111/desc.12086 24118719

[42] Francesca Giardini, Rafael Wittek, editors. The Oxford Handbook of Gossip and Reputation. Oxford University Press; 2019.

[43] Gneezy U. Deception: the role of consequences. Am Econ Rev. 2005 3 1;95(1):384–94.

[44] Sznycer D, Tooby J, Cosmides L, Porat R, Shalvi S, Halperin E. Shame closely tracks the threat of devaluation by others, even across cultures. Proc Natl Acad Sci. 2016 3 8;113(10):2625–30. 10.1073/pnas.1514699113 26903649 PMC4790975

[45] Page R. Saying ‘sorry’: Corporate apologies posted on Twitter. J Pragmat. 2014 2 1;62(Supplement C):30–45.

[46] Blum-Kulka S, Olshtain E. Requests and apologies: A Cross-Cultural Study of Speech Act Realization Patterns (CCSARP)1. Appl Linguist. 1984 10 1;5(3):196–213.

[47] Sznycer D, Schniter E, Tooby J, Cosmides L. Regulatory adaptations for delivering information: the case of confession. Evol Hum Behav. 2015 1 1;36(1):44–51. 10.1016/j.evolhumbehav.2014.08.008 25663798 PMC4313746

[48] Burt RS. Gossip and reputation In: Lecoutre M, Pascal L, editors. Management et réseaux sociaux: ressource pour l’action ou outil de gestion? Paris: Hermes; 2008 p. 27–42.

[49] Feinberg M, Willer R, Stellar J, Keltner D. The virtues of gossip: reputational information sharing as prosocial behavior. J Pers Soc Psychol. 2012 5;102(5):1015–30. 10.1037/a0026650 22229458

[50] Sommerfeld RD, Krambeck H-J, Semmann D, Milinski M. Gossip as an alternative for direct observation in games of indirect reciprocity. Proc Natl Acad Sci. 2007 10 30;104(44):17435–40. 10.1073/pnas.0704598104 17947384 PMC2077274

[51] Fine GA, Rosnow RL. Gossip, gossipers, gossiping. Pers Soc Psychol Bull. 1978 1 1;4(1):161–8.

[52] Giardini F, Conte R. Gossip for social control in natural and artificial societies. SIMULATION. 2012 1 1;88(1):18–32.

[53] Aikhenvald A. Evidentiality. Oxford: Oxford University Press; 2004.

[54] Aksu-Koç AA, Slobin DI. A psychological account of the development and use of evidentials in Turkish In: Chafe W, Nickols J, editors. Evidentiality: The linguistic coding of epistemology. Norwood, NJ: Ablex; 1986 p. 159–67.

[55] Papafragou A. The acquisition of modality: Implications for theories of semantic representation. Mind Lang. 1998 9 1;13(3):370–99.

[56] Papafragou A, Li P, Choi Y, Han C. Evidentiality in language and cognition. Cognition. 2007;103:253–99. 10.1016/j.cognition.2006.04.001 16707120 PMC1890020

[57] Fitneva SA. The role of evidentiality in Bulgarian children’s reliability judgments. J Child Lang. 2008;35(4):845–68. 10.1017/S0305000908008799 18838015

[58] Matsui T, Yamamoto T, McCagg P. On the role of language in children’s early understanding of others as epistemic beings. Cogn Dev. 2006;21:158–73.

[59] Grice HP. Logic and conversation In: Cole P, Morgan JL, editors. Syntax and Semantics. New York: Academic Press; 1975 p. 41–58.

[60] Sperber D, Wilson D. Relevance: Communication and Cognition Cambridge, MA: Harvard University Press; 1986.

[61] Carparo V. Gender differences in lying in sender-receiver games: A meta-analysis. Judgm Decis Mak. 2018;13(4):345–55.

[62] Garbarini F, Boero R, D’Agata F, Bravo G, Mosso C, Cauda F, et al Neural correlates of gender differences in reputation building. PLOS ONE. 2014 9 2;9(9):e106285 10.1371/journal.pone.0106285 25180581 PMC4152267

