# Supplementary material for: "Someone told me": Preemptive reputation protection in communication
Source: PLoS One. 2019 Apr 24;14(4):e0200883. doi: 10.1371/journal.pone.0200883 (PMC6481770; doi:10.1371/journal.pone.0200883)
Supplement: S1 Appendix — Only the Daycare scenario was used in Estonian and Turkish. The scenarios were distributed into four lists according to a Latin Square design for English and Italian participant. (DOCX) [file pone.0200883.s001.docx]

## Giardini, Fitneva, & Tamm: “Someone told me”: Preemptive reputation protection in communication

## SUPPLEMENTARY MATERIAL: Scenarios

Each scenario had an ample and limited resource version (related correspondingly to non-competition and competition). We also manipulated the relationship between the actors: friends vs. acquaintances. Below we show an ample or limited resource version of each scenario in English. We use " / " to show the two relationships.

**1. Concert – limited resource condition**

Sheila finds out from the internet that her favorite rock band is going to play for the first time in her city on the weekend. The concert venue is small and the band is very popular, so she knows tickets will sell out quickly.

Monica is [one of Sheila’s best friends / a student Sheila met once at a party]. She would like to take some friends of hers who are visiting from out of town to a concert. She asks Sheila if there is a good concert on the weekend. Imagine you are Sheila. Which of the following answers are you most likely to give?

a) I just checked online, and there is a great concert coming up this weekend.

b) I just checked online, and I didn’t see anything interesting this weekend.

c) I do not know exactly, but someone told me that there’s nothing interesting this weekend.

d) I do not know exactly, but someone told me that there is a great concert coming up this weekend.

**2. Car - limited resource condition**

David is a first-year graduate student. He needs to buy a car. He has found a car he likes in a small used-car dealership right next to the supermarket where he shops. This dealership is much cheaper than any of the other dealerships he has visited and has a limited inventory of cars. David can’t buy the car right away though because of finances. Matt is [a student David met once at a party/ one of David’s best friends]. He is also looking for a car. He asks David if he knows of a cheap used-car dealer. Imagine you are David. Which of the following answers are you most likely to give?

a) I was looking into car dealers myself recently and I found a very cheap used-car dealership close to the supermarket.

b) I was looking into car dealers myself recently and they were all the same.

c) I do not know exactly, but someone told me that they are all the same around here.

d) I do not know exactly, but someone told me that there is a very cheap used-car dealership close to the supermarket.

**3. Scholarship – ample resource condition**

Brian recently graduated with a degree in Russian Literature and is looking for a scholarship for his graduate studies. He finds out a private foundation that gives a small number of full scholarships. The scholarships are awarded merit-based and there is the same number of awards in the Humanities and the Natural Sciences. Sam is [one of Brian’s best friends / a student Brian met once at a party]. He has a degree in Chemistry and is looking for a graduate scholarship, too. Sam asks Brian if he knows of a source of scholarships. Imagine you are Brian. Which of the following answers are you most likely to give?

a) I checked recently and came across a foundation that gives out scholarships for MA studies. I will email you their contact information.

b) I checked recently a number of foundations but no one offered MA scholarships.

c) I do not know exactly, but someone told me that no one offers MA scholarships.

d) I do not know exactly, but someone told me of a foundation that gives out scholarships for MA studies. I will email you their contact information.

**4. Daycare – ample resource condition**

Mary needs to find a daycare for her daughter Lisa, who is 3. In the neighborhood there are two centers. Mary visits both and finds out that the Blue Center has better equipment, a very friendly staff and a beautiful garden. The Blue Center is new and has lots of spots. [While at the playground one day, Mary meets Margaret who has twins the same age as Lisa. / Mary’s close friend Margaret has twins the same age as Lisa.] Margaret is also looking for daycare and asks Mary which centre she would recommend. Imagine you are Mary. Which of the following answers are you most likely to give?

a) I visited both daycares and the Blue Center is definitely better, you should try to register your kids there

b) I visited both daycares and the Red Center is definitely better, you should try to register your kids there

c) I do not know exactly, but someone told me that the Red Center is definitely better, you should try to register your kids there.

d) I do not know exactly, but someone told me that the Blue Center is definitely better, you should try to register your kids there.
