# Supplementary material for: "Someone told me": Preemptive reputation protection in communication
Source: PLoS One. 2019 Apr 24;14(4):e0200883. doi: 10.1371/journal.pone.0200883 (PMC6481770; doi:10.1371/journal.pone.0200883)
Supplement: S1 Table — (DOCX) [file pone.0200883.s002.docx]

## Giardini, Fitneva, & Tamm: “Someone told me”: Preemptive reputation protection in communication

Effects of Resource Availability and Relationship with the Addressee

on Truthful Responding

Table S1 shows the results of the analyses of the effects of Resource Availability and Relationship on the truthfulness of the at-issue content (i.e., the direct answer to the question). The analyses were conducted using generalized estimating equations (GEE) modeling. Initial analyses showed that scenario did not enter in any interactions in the data from English and Italian speakers, thus it was included as a simple effect in these analyses. Preliminary analyses also showed no effect of gender. Based on model fit statistics, we used GEE with a Poisson log-linear link function to analyze the English and Italian data. (The Poisson distribution approximates the binomial distribution for rare events.) Model fit statistics suggested that binomial models should be used for the analyses of the Turkish and Estonian data.

As Table S1 shows, in three of the studies, speakers were significantly more truthful when resources were ample, i.e., in the absence of competition (see also Figure S1). English and Estonian speakers were also more truthful when responding to a friend's question than an acquaintance's question. The more limited effect of the relationship between the speaker and addressee on the falsification of content could be due to the fact that friends were contrasted with acquaintances rather than strangers. The likelihood of re-encounter with acquaintances may not be negligible. It is also possible that the vignette format of the study was not conducive to eliciting a strong effect of relationship.

The interaction effect between resource availability and relationship for English speakers reflected the greater frequency of false reports when communicating about limited resources to an acquaintance than any of the other thee conditions (all *p*’s < .01).

|  | Language | | | | | |
| --- | --- | --- | --- | --- | --- | --- |
| Effect | English  (N = 80) | Italian  (N = 104) | Turkish  (N = 346) | Estonian  (N = 220) | |  |
| Resource Availability | 5.2* | 1.353 | 11.435* | 7.264** | |  |
| Relationship | 7.729* | 1.229 | 1.757 | 3.083† | |  |
| Resource Availability x Relationship | 6.086* | 1.465 | .096 | .001 | |  |
| Scenario | 12.113* | 6.653† | N/A^a^ | | N/A^a^ | |

Note: Wald chi-square tests significance levels: ** < . 01; * < .05; † < .1.

^a^Just one scenario was used with Estonian and Turkish speakers

Figure S1. Proportion of answers with true content by resource availability and relationship with addressee.
