# Supplementary material for: "Someone told me": Preemptive reputation protection in communication
Source: PLoS One. 2019 Apr 24;14(4):e0200883. doi: 10.1371/journal.pone.0200883 (PMC6481770; doi:10.1371/journal.pone.0200883)
Supplement: S2 Table — (DOCX) [file pone.0200883.s003.docx]

**Giardini, Fitneva, & Tamm: “Someone told me”: Preemptive reputation protection in communication**

Table S2.

*Information Criteria Used in Model Selection Process*

|  | Language | | | |
| --- | --- | --- | --- | --- |
| Effect | English (QICC) | Italian (QICC) | Turkish (BIC) | Estonian (BIC) |
| Full model^1^ | 270.775^ | 371.499 | 74.224^#^ | ^a^ |
| Reduced model^2^ | 269.300 | 371.521 | **68.431** | **57.151**^a^ |
| Reduced model x gender^3^ | 271.076^ | 372.477 | 124.075^##^ | 98.281^a, ##^ |
| Reduced model + gender^4^ | 262.358 | 363.689 | 95.956 | 78.231^a^ |
| Reduced model x scenario^b,5^ | 279.196^ | 368.410 |  |  |
| Reduced model + scenario^b,6^ | **261.396** | **355.010** |  |  |

*Note:* The information criteria used to compare models were the Corrected Quasi Likelihood under Independence Model Criterion (QICC) for the English and Italian data and the Baysian Information Criterion (BIC) for the Turkish and Estonian data. The criteria are in smatter-is-better form. The information criterion values of the models reported in Table 3 in are in bold.

^a^ Estonian speakers always used an indirect evidential when lying to a friend. This led to quasi-complete separation in the data when the Truthfulness x Relationship and the Truthfulness x Relationship x Resource availability terms were included in the models and statistics could not be computed. All reported models excluded these interaction effects. As a result, for Estonian, the Full and Reduced model (1 and 2 below) are equivalent.

b Models including scenario were not run for the Estonian and Turkish data as only one scenario was used in these studies.

^1^ Y = b_0_ + b_1_ * Truthfulness + b_2_ * Resource Availability + b_3_ * Relationship +b_4_ * Truthfulness * Resource Availability + b_5_ * Truthfulness * Relationship + b_6_ * Truthfulness * Relationship * Resource Availability

^2^ Y = b_0_ + b_1_ * Truthfulness + b_2_ * Resource Availability + b_3_ * Relationship +b_4_ * Truthfulness * Resource Availability + b_5_ * Truthfulness * Relationship

^3^ Y = b_0_ + b_1_ * Truthfulness + b_2_ * Resource Availability + b_3_ * Relationship +b_4_ * Truthfulness * Resource Availability + b_5_* Truthfulness * Relationship + b_6_ * gender + b_7_ * Truthfulness * gender + b_8_ * Resource Availability * gender + b_9_ * Relationship * gender + b_10_ * Truthfulness * Resource Availability * gender + b_11_ * Truthfulness * Relationship * gender

^4^ Y = b_0_ + b_1_ * Truthfulness + b_2_ * Resource Availability + b_3_ * Relationship +b_4_ * Truthfulness * Resource Availability + b_5_ * Truthfulness * Relationship + gender

^5^ Y = b_0_ + b_1_ * Truthfulness + b_2_ * Resource Availability + b_3_ * Relationship +b_4_ * Truthfulness * Resource Availability + b_5_* Truthfulness * Relationship + b_6_ * scenario + b_7_ * Truthfulness * scenario + b_8_ * Resource Availability * scenario + b_9_ * Relationship * scenario + b_10_ * Truthfulness * Resource Availability * scenario + b_11_ * Truthfulness * Relationship * scenario

^6^ Y = b_0_ + b_1_ * Truthfulness + b_2_ * Resource Availability + b_3_ * Relationship +b_4_ * Truthfulness * Resource Availability + b_5_ * Truthfulness * Relationship + scenario

Introducing higher level (3-way) interaction effects led in most cases to model instability and remediation of the problems (listed below) was not found. It should be noted that: 1) we had no theoretical interest in the variables of Gender and Scenario; these analyses were initiated as standard methodological checks and 2) the significance of the effects (at/different from chance) reported in Table 3 was consistent across models.

^ Hessian matrix is singular.

# The maximum number of step-halvings was reached but the log-likelihood value cannot be further improved.

## A quasi-complete separation may exist in the data.
