# Supplementary material for: "Someone told me": Preemptive reputation protection in communication
Source: PLoS One. 2019 Apr 24;14(4):e0200883. doi: 10.1371/journal.pone.0200883 (PMC6481770; doi:10.1371/journal.pone.0200883)
Supplement: S1 Fig — (DOCX) [file pone.0200883.s004.docx]

## Giardini, Fitneva, & Tamm: “Someone told me”: Preemptive reputation protection in communication

**S1 Fig.**
